# Supplementary material for: Incidence of COVID-19 reinfection among Midwestern healthcare employees
Source: PLoS One. 2022 Jan 4;17(1):e0262164. doi: 10.1371/journal.pone.0262164 (PMC8726474; doi:10.1371/journal.pone.0262164)
Supplement: Attachment — Submitted filename: Resonse to Reviewers 12.16.2021.docx [file pone.0262164.s001.docx]

Rebuttal Letter

| Editor’s Comments | Author’s Comment |
| --- | --- |
| INTRODUCTION:  I understand the Author's point of view. However, I abide by my previous comment. The introduction should highlight the needing for evidence in this topic in this specific historical moment. Therefore, it should be highlighted the health system burden due to medical care and disabling sequelae, mentioning the role of post-COVID therapies/behavioral modifications in the lifestyle change and risk of recurrence. According to this, you should cite the following references: • Curci C et al. Functional outcome after inpatient rehabilitation in post intensive care unit COVID-19 patients: findings and clinical implications from a real-practice retrospective study. Eur J Phys Rehabil Med. 2021;57(3):443-450. doi:10.23736/S1973-9087.20.06660-5 • Iddir M et al. Strengthening the Immune System and Reducing Inflammation and Oxidative Stress through Diet and Nutrition: Considerations during the COVID-19 Crisis. Nutrients. 2020;12(6):1562. Published 2020 May 27. doi:10.3390/nu12061562 • Ferraro F et al. COVID-19 related fatigue: Which role for rehabilitation in post-COVID-19 patients? A case series. J Med Virol. 2021;93(4):1896-1899. doi:10.1002/jmv.26717 • Andrenelli E et al. Systematic rapid living review on rehabilitation needs due to COVID-19: update to May 31st, 2020. Eur J Phys Rehabil Med. 2020;56(4):508-514. doi:10.23736/S1973-9087.20.06435-7 | Thank you for this suggestion. As stated earlier, this study is strictly an epidemiological look at reinfection with no information on symptoms, treatments or interventions. This is not a paper on the burden of COVID nor do we have any data to support the role of therapies or modifications in the prevention of recurrence. However, we added your resources and the need for research on the relationship between lifestyle changes, like behavioral modifications and post-COVID therapies, and risk of recurrence in the Discussion section. |
| METHODS. The number of patients enrolled in a prospective study represents a study result. Please present these data in the Results section rather than the Methods Section. | The only section of the Methods that describes number of patients is in the “Participants” sub-section in order to provide context, as this study’s sample was drawn from a larger prospective study. The number of patients enrolled in the *larger* prospective study is described in the Methods. *This* study’s number of patients is provided in the Results section, as you suggested and we agree that it represents a study result. |
| DISCUSSION. Please discuss the huge difference in percentage between females and males probably because women are much, much more likely to be in clinical roles in a healthcare setting – typically as nurses or medical assistants. | Thank you for this suggestion. The Discussion section has been updated to reflect this. |
| RESULTS AND DISCUSSION. This section should be divided into two different sections. The manuscript form is not suitable for the journal without the suggested improvements. | The Discussion section is a separate sub-section within the Results and Discussion heading, per PLOS ONE’s style requirements as provided to us in this link: [https://journals.plos.org/plosone/s/file?id=wjVg/PLOSOne_formatting_sample_main_body.pdf](https://urldefense.com/v3/__https:/journals.plos.org/plosone/s/file?id=wjVg*PLOSOne_formatting_sample_main_body.pdf__;Lw!!PrVBqlTvcBbYrqSF!RSnPBrNk2V4ogYxBbMpnSnFGiyD7JDrPFlrCp09kAYmNddhTsDxjOwXADeAjwMs$) |
